# Response to the letter by Blank *et al*. regarding the article: ‘LINAC-Based STereotactic Arrhythmia Radioablation (STAR) for paroxysmal atrial fibrillation in elderly: a prospective phase II trial’

**DOI:** 10.1093/europace/euae197

**Published:** 2024-07-11

**Authors:** Antonio Di Monaco, Alba Fiorentino, Massimo Grimaldi

**Affiliations:** Department of Cardiology, General Regional Hospital ‘F. Miulli’, Acquaviva delle Fonti, Bari, 70021, Italy; Department of Radiation Oncology, General Regional Hospital ‘F. Miulli’, Acquaviva delle Fonti, Bari, Italy; Department of Medicine, LUM University, Casamassima, BA, Italy; Department of Cardiology, General Regional Hospital ‘F. Miulli’, Acquaviva delle Fonti, Bari, 70021, Italy

We greatly appreciate your interest in our experimental study^1^ and agree with all considerations^2^; however, some points deserve further discussion.

## Selection of patients

Atrial fibrillation (AF) prevalence in the elderly is growing up, and in Europe, in 2060, the number of patients older than 75 years with AF is estimated to be 13.8 million.^3^ Although AF is considered a ‘benign’ arrhythmia, recent data with long-term follow-up showed that increased time in sinus rhythm was associated with a decrease in mortality and adverse events.^4^ Elderly patients are frail, and AF is difficult to treat with drugs due to the frequent atrioventricular node conduction or intraventricular conduction delays or due to tachy-brady syndrome.^3,5^ Ablation of the atrioventricular node and pacemaker implantation can control ventricular rate, exposing the patient to device malfunction and infection risk.^5^ Catheter ablation in elderly patients carries a higher risk of complications, despite the use of safer energy sources.^6,7^ Furthermore, the notable operator dependence inherent in the ablation is highlighted. To answer the question of which approach, between radiotherapy and ablation, is less risky, a randomized study would be necessary. Thus, our study was focused on safety for the elderly (median age of 77 years) highly symptomatic and unresponsive to drugs.

## Safety of AF-STAR

We agree with Blank *et al*. about the risk of long-term side effects in a benign condition and the precision of RT. Due to the latter consideration, we evaluated the safety aspect of our trial, performing a ‘simultaneous integrated protection’ dose for the oesophagus and bronchus to reduce the risk of side effects. A previous publication reported an oesophago-pericardial fistula and death after six months from STAR for a recurrent VT.^8^ The patient received more therapies before Cyberknife-STAR (arterial coronary revascularization, including gastroepiploic artery and a catheter ablation). Cyberknife has several differences to LINAC.^9^ Linac-STAR treatment time was very short (3 min), reducing the risk of oesophagus displacement during treatment,^10^ and it has the possibility to live evaluate organ movements. Moreover, in our trial, all patients were naïve, excluding patients who had undergone previous catheter ablations.

## Side effects

We reported the one-month safety and all the possible events related to STAR. No acute toxicity more than grade 3 related to irradiation occurred. Regarding the grade 4 event, as reported, it is impossible to determine if it was due to STAR or chance.

Finally, although the study focused on safety, we demonstrated that STAR effectively performs pulmonary vein isolation, improving quality of life.^1^

In conclusion, as Blank *et al*. reported, we confirmed that a long-term follow-up is needed.

However, STAR is a promising treatment for AF elderly patients in the absence of other treatment strategies. The outpatient care setting is of great importance, considering the large number of patients eligible for the procedure instead of the progressive reduction of hospital beds and the need to reduce healthcare spending.

In the future, after assessing long-term safety, we intend to plan a trial focused on efficacy, comparing STAR with catheter ablation or the ‘ablate and pace’ strategy. New RT techniques, and artificial intelligence for image processing, may improve, in the near future, AF-treatment scenario.
